# Environmental impact of cataract surgery and strategies for mitigation: a life cycle assessment in an academic hospital

**DOI:** 10.1097/j.jcrs.0000000000001777

**Published:** 2026-09-02

**Authors:** Emma J. Kooistra, Laura Golsteijn, Egid M. van Bree, Sjoerd Elferink, Evelyn A. Brakema, Floris Hermeling, Pleun Hemelaar, Anne Ottenbros, Niels Crama, Rosalie van Zelm, Hugo R.W. Touw, Tim Stobernack

**Affiliations:** From the Department of Intensive Care Medicine, Radboud University Medical Center, Nijmegen, the Netherlands (Kooistra, van Bree, Hermeling, Hemelaar, Touw, Stobernack); Center for Acute and Intensive Care, Radboud University Medical Center, Nijmegen, the Netherlands (Kooistra, Stobernack); PRé Sustainability, Amersfoort, the Netherlands (Golsteijn); Department of Ophthalmology, Flevoziekenhuis, Almere, the Netherlands (Elferink); Department of Public Health and Primary Care, Leiden University Medical Centre, Leiden, the Netherlands (Brakema); Department of Environmental Sciences, Radboud Institute for Biological and Environmental Sciences, Radboud University, Nijmegen, the Netherlands (Hermeling, Ottenbros, van Zelm); Department of Ophthalmology, Radboud University Medical Center, Nijmegen, the Netherlands (Crama).

## Abstract

**Purpose:**

To identify the main environmental hotspots of cataract surgery and evaluates strategies to reduce its environmental impact and to compare process-based and monetary value-based methodologies for environmental assessment.

**Setting:**

Radboud University Medical Center, Nijmegen, the Netherlands.

**Design:**

Cradle-to-grave life cycle assessment (LCA) following ISO 14040/44 guidelines.

**Methods:**

The environmental impact of phacoemulsification cataract surgery was assessed, including all processes, products, and equipment used in the operating room. Key contributors to human health and ecosystem damage were identified. A monetary value-based analysis was performed using the same inventory data to compare methodological approaches.

**Results:**

Disposables (35%) and patient travel (31%) were the largest contributors to the environmental impact. Immediate sequential bilateral cataract surgery (ISBCS) reduced the impact by 19%, and simplifying surgical kits achieved a 10% reduction. Additional reductions were observed with renewable energy (−12%) and sustainable transportation (−8%). The monetary value-based approach yielded a fourfold higher carbon footprint than the process-based LCA, while identifying similar hotspots.

**Conclusions:**

The main environmental hotspots of cataract surgery were patient travel and disposable products. The most effective mitigation strategy to reduce the carbon footprint is implementing ISBCS. Additional mitigation strategies include reducing the use of disposable products, encouraging sustainable travel options, and switching to renewable energy. These findings highlight opportunities for ophthalmologists to contribute to more sustainable surgical care.

## Abstract 2

This study identifies disposables and patient travel as key environmental hotspots in cataract surgery and demonstrates that targeted strategies such as ISBCS and kit simplification can reduce the carbon footprint substantially.

Today's modern healthcare and continuous medical developments aim to ensure human well-being. At the same time, the healthcare sector accounts for 4.4% of the global carbon footprint, and in the Netherlands, it is responsible for 7.3% of the national carbon footprint and 13.0% of resource extraction.^1,2^ Environmental degradation endangers the health of our planet and affects all facets of human health, including infectious and cardiovascular diseases, mental health, and ocular diseases.^3–5^ Specifically in ophthalmology, the expected impacts of climate change include increasing incidence rates of trachoma infections, cataract, severe allergic eye diseases, glaucoma, and age-related macular degeneration.^6–10^

Cataract surgery is one of the most commonly performed and cost-effective procedures worldwide, particularly since the introduction of phacoemulsification.^11^ Given the high volume of cataract surgeries, assessing and reducing its environmental impact can substantially contribute to more sustainable ophthalmic care. To date, studies examining the environmental impact of cataract surgery solely focused on the carbon footprint, neglecting other environmental factors.^12–15^ Furthermore, methodological differences (eg, monetary value-based vs process-based approaches) have resulted in a wide range of reported outcomes and reduced generalizability.^16^

Environmental impact analyses assess the impact that a product or process has on the environment, such as carbon footprint, air pollution, and water use. The routine method to investigate the environmental impact of products or processes is life cycle assessment (LCA). In a process-based LCA, each step of a product or service's life cycle is analyzed in detail—from raw material extraction to disposal—by measuring all relevant material and energy inputs and the associated emissions. Methods for LCAs have been standardized in the ISO-14040 and ISO-14044 guidelines.^17^ Alternatively, monetary-based methods estimate environmental impact by linking financial costs to environmental impact using average conversion factors. Although this approach is faster and can include broader upstream effects (eg, general manufacturing overhead), it has a limited level of detail and is mainly suitable for the analysis of entire healthcare systems. By contrast, process-based LCA provides more precise, product-specific insights, making it more appropriate for analyzing individual procedures.

Using environmental impact analyses, key contributors to the environmental impact—“environmental hotspots”—can be identified for effective mitigation strategies. In this LCA study, we primarily aim to assess the “environmental impact hotspots” of cataract surgery to develop effective mitigation strategies. Furthermore, we compare results of process-based LCA and monetary value-based methodology.

## METHODS

### Study Design

A single-center LCA was executed from May until November 2022 in the Radboud University Medical Centre (Radboudumc) in the Netherlands. Patients requiring cataract surgery of both eyes were treated on 2 separate days, according to hospital protocols. Similar to previously published LCA studies in health care, 10 adult patients who underwent isolated cataract surgery by phacoemulsification were included to allow for variability of resource consumption.^18,19^ No further inclusion or exclusion criteria were formulated. The study was confirmed as non–medical-scientific research by the Medical Research Ethics Committee Oost-Nederland (2021-13265). Data are presented as median with interquartile ranges (IQRs).

### Life Cycle Assessment

Following international guidelines (ISO-14040/44), this LCA consisted of 4 stages: (1) defining the goal and scope of the study; (2) the life cycle inventory (LCI) phase—involving data collection; (3) life cycle impact assessment, during which the environmental impact of collected data is characterized for multiple impact categories; and (4) the interpretation phase.^17,20,21^

### Goal and Scope

**Figure 1.**
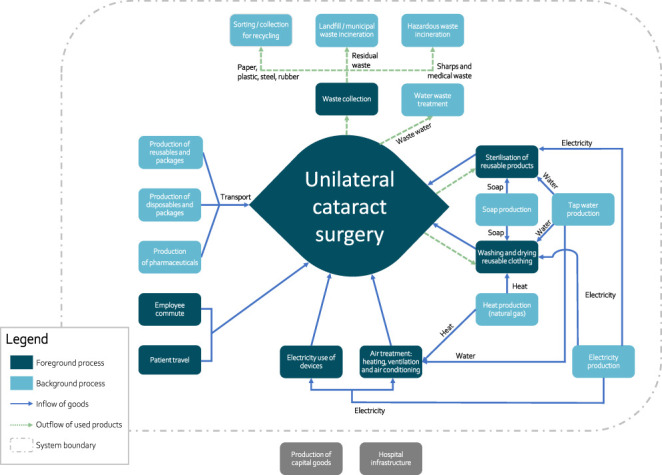
System boundaries for the life cycle assessment of cataract surgery. Processes in gray were outside the system boundaries.

The subject of study (functional unit) was the performance of cataract surgery during which an intraocular lens (IOL) was removed by phacoemulsification and replaced by an IOL implant in 1 eye. The LCA included the entire life cycle of all required resources, from the extraction of raw materials to waste/recycling processes (“cradle to grave”) (Figure 1). The total operating room (OR) time was defined as the duration from the patient's entry into the OR until exit, encompassing all preoperative and postoperative activities, in addition to the surgical procedure itself. Processes or products of which the environmental impact was expected to be negligible were not included in the study, such as the production of capital goods (eg, microscope and screens) and hand washing. Resources applying to multiple patients were allocated based on size, mass, or time (eg, laundry processes and staff commute).

### Life Cycle Inventory

Foreground data were obtained through process observations, waste audits, electronic patient files, literature, and through manufacturers and process experts. During observations in the OR, inventory lists served as a tool for scoring and documenting the usage of specific products. Product weight and material compositions were determined. Waste audits were executed to assess the amount of waste. Dosages of administered pharmaceuticals were identified from electronic patient files. In case data for medical devices, materials, and pharmaceuticals were not available, assumptions were made based on expert consultations, the waste processor, and literature research. All used products and processes were categorized into 8 subcategories: disposables, reusables, patient travel, employee commute, energy use, laundry processes, pharmaceuticals, and the IOL implant. All data were anonymously stored in an inventory list in Microsoft Excel (Microsoft Corp.). Background data were obtained using the STREAM database by CE Delft for transport data within the Netherlands and the ecoinvent database (ecoinvent 3.9) for all other products and processes.^22–24^ Detailed methodology of the LCI phase and subcategories is described in Table 1 and Supplemental Methods (available at http://links.lww.com/JRS/B459).

**Table 1.** Overview of inventory data collection per subcategory

Subcategory	Description of subcategory	Details of data collection
Disposables	All disposable products used during cataract surgery, such as surgical gowns, medical gloves, surgical drapes, and gauzes	Type and quantities were determined based on interviews with surgical assistants, observations in the OR, and waste audits
Reusables	Tray of surgical instruments used during cataract surgery	Type and quantities of the instruments were determined based on interviews with surgical assistants and observations in the OR. Details of washing and sterilization processes were based on the literature and information from the local sterilization department
Pharmaceuticals	Administered pharmaceuticals during the surgical procedure	Information about dosages of administered pharmaceuticals was anonymously obtained from the electronic patient files
IOL implant	The lens implanted during cataract surgery	Information about the type of lens was obtained through surgical assistants and observations in the OR. Material compositions were obtained through online information of the manufacturer
Energy use	Energy use in the OR by HVAC, lights, and devices	Energy use was based on data of the local engineer. Dutch energy mix (STREAM) was used for the main analysis
Laundry processes	Laundry of staff clothing	Type and quantities of used clothes were based on observations in the OR. Details of laundry processes were obtained from the external laundry company
Patient travel	Travel distances of patients from home to the hospital and back on the day of surgery	Distances were calculated based on information in the electronic patient files. It was assumed that all patients travelled by an average Dutch car without detours. Travel related to preoperative or postoperative appointments was not included
Employee commute	Travel distances of employees from home to the hospital and back	Distances and means of transport were inventoried through interviews with staff in the OR

HVAC = heating, ventilation, and air conditioning; OR = operating room

### Life Cycle Impact Assessment

Median values and IQRs of inventoried data were entered in SimaPro 9.5.0.1 LCA-software (PRé Sustainability) using processes of the ecoinvent 3.9 database (Used background processes of inventory processes in SimaPro software are available on request.).^24^ Environmental impacts were calculated with the ReCiPe 2016 v. 1.1 method (Midpoint [H] and Endpoint [H]).^25^ This method includes a total of 18 midpoint indicators, such as global warming (the carbon footprint), which can be aggregated into endpoint indicators: damage to human health and damage to ecosystems. We presented outcomes as absolute values in ReCiPe reporting units, such as kilograms carbon dioxide equivalents (kg CO_2_eq) and percentages of human health damage and ecosystem damage. Owing to limited data availability, pharmaceuticals were only considered for the impact of the associated carbon footprint.

### Interpretation Phase

Midpoint indicators contributing ≥10% to human health and/or ecosystem damage were described in detail throughout the study. For each of these indicators, the contribution of the 8 aforementioned procedural subcategories was ranked based on their share of the total environmental impact. Those subcategories contributing most to 1 or multiple indicators were considered “environmental hotspots.”

The influence of database choices, assumptions, and contextual variations (eg, patient travel distance) was evaluated in a sensitivity analysis. First, variability in patient and employee travel distances was quantified by adjusting travel distances to 70% and 130% (transport 70% and transport 130% scenarios). Second, the sensitivity of the results was tested by using energy and transport data from the ecoinvent 3.9 database instead of the STREAM data (full ecoinvent scenario).^22–24,26^ Third, to reflect potential underestimation of pharmaceutical's impact, a 30% increase of use was calculated (pharmaceuticals 130% scenario).

Uncertainties in measurement accuracy of foreground data and representativeness of background data were computed using a pedigree matrix for data quality. Ranges were converted into 95% CIs for the midpoint environmental impacts using Monte Carlo simulations of 10 000 runs in the SimaPro LCA software.^27,28^

### Mitigation Scenarios

Based on the environmental hotspots identified in the main analysis, multiple hypothetical scenarios were developed to determine effective mitigation strategies. We accounted for the fact that most patients require cataract surgery in both eyes, typically performed as delayed sequential surgery, where the operations are conducted consecutively rather than simultaneously. Also, based on expert review (by S.E., ophthalmologist) and the ESCRS sustainable Cat-pack (results unpublished), a scenario using a simplified inventory list with only strictly necessary materials was proposed as a mitigation strategy.^29^

### Monetary Value-Based Analysis

To compare results of different methodologies, an additional impact assessment was executed wherein prices were converted into environmental impact using DEFRA's and “Bilan Carbone” emission conversion factors.^12,14^ Methods of this monetary value-based analysis were described in Supplemental Methods (available at http://links.lww.com/JRS/B459) and were comparable with methods performed by Morris and colleagues and by Ferrero and colleagues.^12,14^

## RESULTS

### Procedure Characteristics

Ten patients were included from May until November 2022. Surgical procedures lasted for 25 (17 to 36) minutes, contributing to a total time spent in the OR of 59 (41 to 60) minutes per patient.

### Inventory Data

A team of 4 (4-4) employees were directly involved per cataract surgery and travelled 44 (7 to 68) km daily (58% of the kilometers were travelled by car, 36% by electric car, 3% by bicycle, and 3% by electric bicycle). Patient's travel distance from their residence to the Radboudumc and back by car was 40 (28 to 68) km.

The flow of all used products during cataract surgery at the Radboudumc is depicted in Figure 1. A total of 61 (55 to 67) disposable products was used, leading to 2.5 (2.4 to 2.9) kg waste per cataract surgery procedure. A picture of all materials used during cataract surgery at the Radboudumc is shown in Supplemental Figure 1 (available at http://links.lww.com/JRS/B460). A list including all inventoried products and processes is provided in Supplemental Data 1 (available at http://links.lww.com/JRS/B455).

### Life Cycle Assessment

**Figure 2.**
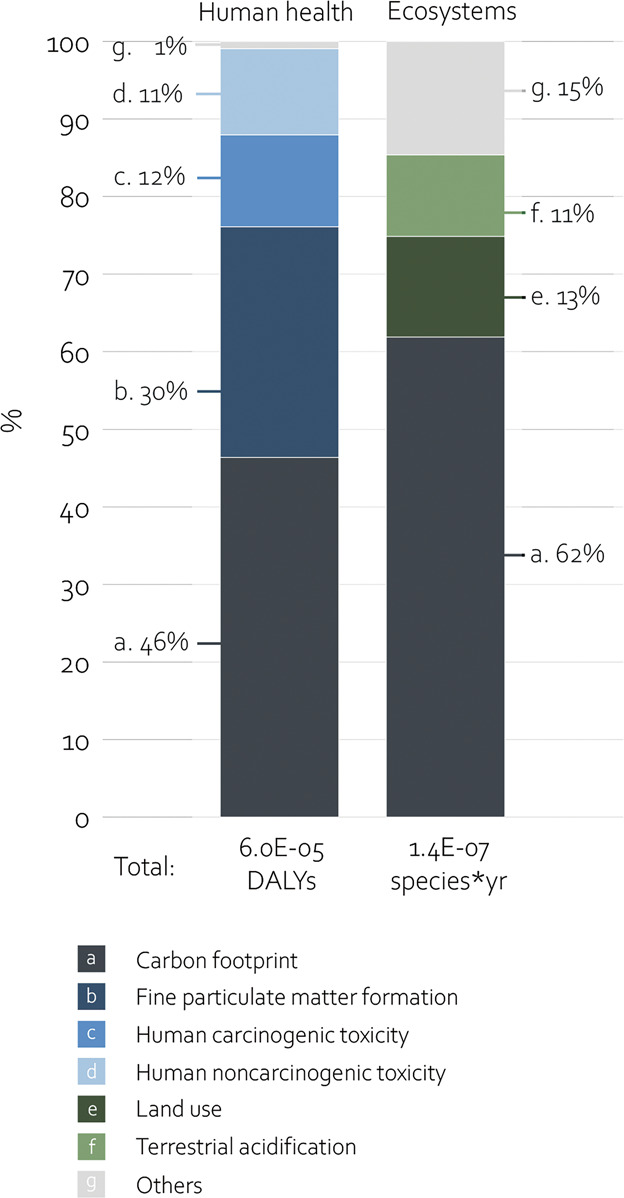
Environmental impact of cataract surgery. Bar graphs presenting the contribution of midpoint indicators to damage to human health and ecosystems in percentage. Midpoint indicators can contribute to both human health damage and ecosystem damage or to either one. Midpoint indicators contributing <10% were described as “others.” DALY = disability-adjusted life year

The environmental impact of cataract surgery led to human health damage of 6.0E-5 (4.4E-5 to 8.0E-5) DALYs and ecosystem damage of 1.4E-7 (1.0E-7 to 1.8E-7) species × year. The carbon footprint (30.0 [22.3 to 38.8] kg CO_2_ eq) was the primary environmental factor impacting both human health damage (46%) and ecosystem damage (62%) (Figure 2). Furthermore, fine particulate matter formation of 0.03 (0.02 to 0.04) kg PM2.5 eq, human carcinogenic toxicity of 2.1 (1.4 to 3.0) kg 1.4-DCB eq, and human noncarcinogenic toxicity of 29.3 (21.1 to 39.5) kg 1.4-DCB eq were the main midpoints adversely affecting human health (Figure 2). Regarding damage to ecosystems, also land use (2.0 [1.5 to 2.6] m^2^a crop eq) and terrestrial acidification (0.07 [0.05 to 0.09] kg SO_2_ eq) contributed at least 10% to the total impact. An overview of the contribution of all midpoint indicators to endpoints is presented in Figure 2 and Supplemental Table 1 (available at http://links.lww.com/JRS/B462).

### Environmental Hotpots

**Figure 3.**
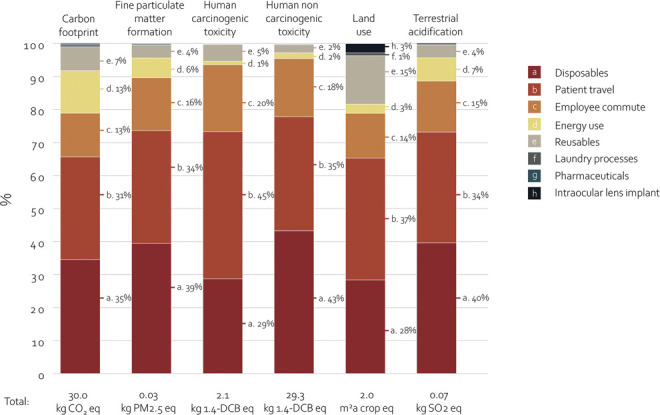
Contribution of subcategories to the environmental impact of cataract surgery. Contribution of disposables, reusables, pharmaceuticals, IOL implant, energy use, laundry processes, patient travel, and employee commute were presented in % for the 6 main midpoints. For pharmaceuticals, impacts were quantified on the carbon footprint only.

**Figure 4.**
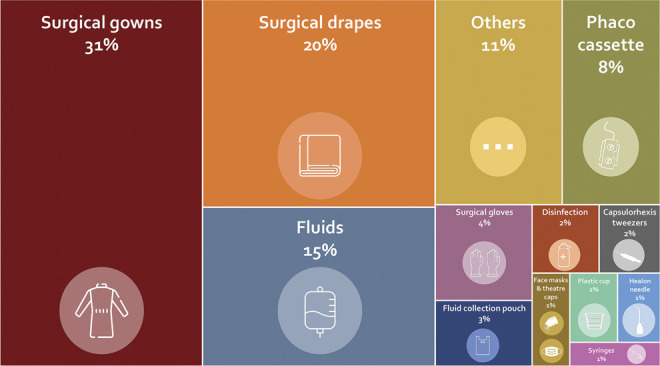
Contribution of individual disposable products to the carbon footprint of the disposables subcategory. The disposables subcategory was responsible for the emission of 10.4 kg CO_2_eq, which was 35% of the overall carbon footprint of cataract surgery.

Disposables were responsible for 35% of the carbon footprint of cataract surgery, with 3 surgical gowns per procedure being the main contributor among them (Figure 3 and Supplemental Table 2, available at http://links.lww.com/JRS/B463). The contribution to the carbon footprint of all individual disposable products within the disposables subcategory is depicted in Figure 4. Other main contributing subcategories were patient travel, employee commute, and energy use, accounting for 31%, 13%, and 13% of the total carbon emissions, respectively. The distribution of subcategory contributions to other environmental impact midpoints followed a similar pattern (Figure 3 and Supplemental Table 2, available at http://links.lww.com/JRS/B463). Notably, in comparison with the carbon footprint, the reusable materials had a proportionally higher contribution to land use. This was primarily attributed to the sterilization papers needed for packaging of sterilized reusable items.

### Mitigation Strategies

Based on the identified environmental hotspots, several hypothetical mitigation scenarios were developed (Table 2). The largest reductions were obtained by targeting patient travel. Immediate sequential bilateral cataract surgery (ISBCS) for patients requiring surgery in both eyes resulted in a reduction of 5.7 kg CO_2_eq per eye, a 19% decrease compared with delayed sequential surgery. Furthermore, the carbon footprint could be reduced by running the hospital entirely on renewable energy (−12%) and by replacing employees' car commute with train travel (−8%) or electric cars (−4%). In addition, after an expert evaluation by an ophthalmologist (S.E.), the surgical set was simplified by removing unnecessary disposable and reusable items, leading to a 10% reduction in the carbon footprint. Finally, using 1 surgical gown less resulted in a 4% reduction of the carbon footprint. The list of the products in the simplified surgical kit can be found in Supplemental Data 2 (available at http://links.lww.com/JRS/B456).

**Table 2.** Effects of multiple mitigation strategies calculated based on carbon footprint hotspots

Mitigation strategy	Carbon footprint (kg CO_2_eq)	Reduction compared with original scenario (kg CO_2_eq (%))
ISBCS: per eye^a^	24.3	5.7 (19)
Energy use: use of Dutch green energy mix	26.5	3.5 (12)
Disposables and reusables: simplified surgical set^b^	26.8	3.1 (10)
Employee commute: cars switched to train^c^	27.5	2.4 (8)
Employee commute: combustion engine cars switched to electric cars^d^	28.7	1.3 (4)
Disposables: reduction of 1 surgical gown per procedure	28.7	1.2 (4)

ISBCS = immediate sequential bilateral cataract surgery

Effects were expressed in kg CO_2_ equivalents and the percentage of the total carbon footprint of cataract surgery

a Due to higher efficiency when performing ISBCS compared with unilateral surgery, a reduction of 13% was considered for employee commute, energy use, and employee clothing based on the literature.^30^ Patient travel was reduced by 50%. The amounts of disposables and reusables were unchanged.

b Based on an expert evaluation, the disposable and reusable inventory list has been reduced to only the strictly necessary products. In this scenario, 50% less reusable products were included (divided over 0.5 DIN), 10 disposable products were removed, and the size of 2 drapes was reduced.

c The initial distances for traveling by car have been replaced by train; however, no consideration has been given to actual possibilities for train travel.

d The average Dutch energy mix was taken for charging the electric cars.

### Sensitivity and Uncertainty Analyses

Across the various scenarios in the sensitivity analysis, a carbon footprint range of −13% to +31% was observed. Notably, the environmental hotspots remained consistent. The uncertainty analysis revealed a 95% CI in carbon footprint of 26.4 to 33.7 kg CO_2_eq. Detailed results of the sensitivity analysis and uncertainty analysis are provided in Supplemental Data 3 (available at http://links.lww.com/JRS/B457).

### Monetary Value-Based Analysis

The monetary value-based analysis resulted in a carbon footprint of 125.7 kg CO_2_eq per cataract surgery. Taken together, disposables and waste treatment were responsible for 51% of the overall calculated carbon footprint. Other main contributors were the IOL and pharmaceuticals, accounting for 18% and 11% of the carbon footprint, respectively. To compare results of the process-based LCA and component analysis, contribution of different subcategories to carbon footprint of cataract surgery was presented in Supplemental Figure 2 (available at http://links.lww.com/JRS/B461). Energy use, reusables, and travel movements of both patients and employees contributed proportionally less in the component analysis compared with the process-based LCA.

## DISCUSSION

The carbon footprint, estimated at 30.0 kg CO_2_eq, was the largest contributor to the overall environmental impact of cataract surgery. Other major environmental impact factors were fine particulate matter formation, human (non)carcinogenic toxicity, land use, and terrestrial acidification. Disposable products and patient travel were the major environmental hotspots, providing targets for mitigation strategies. Regarding waste, a total of 2.5 kg was produced per cataract surgery.

Based on the identified environmental hotspots, we recommend various mitigation strategies. First, to address the impact of patient travel, we propose ISBCS. Although bilateral cataracts are typically treated on separate days, requiring multiple travel movements, a recent landmark study demonstrated that ISBCS is both safe and cost-effective.^31^ We acknowledge that ISBCS may not be suitable for all patients or surgeons, for example due to preferences regarding IOL selection based on the first-eye outcome. ISBCS halves patient travel movements, but it does not mitigate the other hotspot: the use of disposables. This is because ISBCS is currently considered as 2 independent procedures due to infection prevention regulations. However, in Aravind Eye Care System's in India, multiple ISBCSs are performed using the same set of materials, with intermediate use of an autoclave in the OR, leading to less need for fully wrapped sterilized sets and a very low postoperative infection rate.^15^ Whether this method can be safely applied in Western countries in accordance with applicable infection prevention measures could be considered. Another considerable measure to reduce disposable products is switching to reusable variants. A previously published study reported a reduction of 66% in CO_2_ emissions and even a 83% reduction of water consumption when using reusable gowns instead of disposable ones.^32^ In addition, we tested a mitigation scenario inspired by the sustainable Cat-pack proposed by the ESCRS (results unpublished).^29^ Based on expert evaluation, we simplified the surgical kit to include only strictly necessary disposables and reusables, resulting in a 7% reduction in the total carbon footprint. This suggests that streamlining surgical kits, though requiring careful consideration, can be a valuable and practical step in reducing the environmental impact of surgical care in general. Furthermore, our study demonstrated that encouraging sustainable transportation methods for patients and employees—such as bicycles, public transport, or electric cars—can also substantially mitigate the environmental impact of health care. Finally, next to reducing the environmental impact of cataract surgery itself, prevention of cataract will be the most effective strategy for minimizing the environmental impact. Numerous risk factors are associated with the development of cataract, such as direct exposure to UV rays, diabetes mellitus, smoking, and cardiovascular diseases.^33,34^ In addition to the recommendations based on this study, there are already many initiatives worldwide to make ophthalmic care more sustainable. For example, much information about this can be found on www.eyesustain.org.

Previously published studies showed substantial variation in the carbon footprint of cataract surgery.^12–15,35^ For example, an international study using the Eyeficiency tool across nine sites estimated emissions between 41 and 130 kg CO_2_eq per surgery, which is 1.5 to 4.5 times higher than our findings.^35^ These differences are mainly due to variations in methodology. Although we conducted a process-based LCA in 1 academic hospital, this study combined a process-based LCA (covering electricity use, patient and staff transportation, reusables production and sterilization, and waste management) with an environmentally extended input–output LCA for disposables, across different countries. Similarly, a study in the United Kingdom documented a carbon footprint 6 times higher than in our study.^12^ Although they considered similar subcategories, this study by Morris and colleagues was not a process-based LCA. Instead, they applied emission factors to convert inventory data into the carbon footprint. To explore the effects of variation in methods, we performed a monetary value-based analysis similar to the approach used by Morris and colleagues.^12^ This analysis indicated a 4 times larger carbon footprint compared with the results of our process-based LCA. The discrepancy highlights how cost-intensive items, such as the IOL implant and medications, emerge as hotspots in monetary value-based analyses due to their high price, which reflects production, quality control, and research and development efforts. Our process-based LCA estimated the environmental impact of the IOL implant based on its plastic content, production, and transport using ecoinvent and STREAM databases in SimaPro software. These findings underscore the challenges of comparing studies with different methodologies. A process-based LCA is considered more reliable because it uses detailed background data for each product and process, breaking down each component (eg, materials in a product) to provide a more accurate and comprehensive assessment. General conversion factors in monetary value-based methods provide broader estimates. However, process-based LCAs do not include impacts such as quality control and research and development efforts, which are indirectly reflected in monetary value-based analyses through the higher costs.

This study has several limitations. First, when data were not available, assumptions about material compositions were made. This could lead to both an overestimation and an underestimation of the environmental impact of individual components of cataract surgery. However, sensitivity and uncertainty analysis revealed that the identified environmental hotspots of cataract surgery will not be significantly affected by the assumptions that were made. Second, the exact environmental impact of the pharmaceuticals used during cataract surgery is unknown. Although some data are available regarding the carbon footprint associated with certain drugs, the environmental impact of medications commonly used in cataract surgery has not been investigated. For the pharmaceuticals of which the carbon footprint was unknown, proxies were applied based on available data for other drugs. Consequently, the estimated environmental impact of medication in this study represents a rough approximation and might be an underestimation. Nevertheless, since ophthalmological procedures require a few drops of medication, this will not be one of the environmental hotspots of cataract surgery, which was supported by the lack of changes in results after increasing pharmaceutical use by 30% in the sensitivity analysis. At the studied Dutch academic center, single-dose units (minims) were used for topical mydriatics and anesthetics. However, practices differ substantially worldwide, as shown by surveys in North America and Europe indicating that nearly half of respondents use multidose bottles across multiple patients, whereas others discard them after a single use.^30,36^ In some countries, including the United States, many institutions require that multidose bottles of dilating drops be discarded after a single use, leading to significant avoidable waste and increased environmental impact. This practice has been shown to incur substantial financial and environmental costs and is more common in hospital settings compared with freestanding surgery centers.^36^ Reducing unnecessary pharmaceutical waste—by enabling safe reuse of multidose bottles in accordance with infection control guidelines—represents a modifiable target for sustainability in cataract surgery, particularly in settings where such waste is prevalent. Guidance on such measures is provided in the multisociety Eyesustain position paper on reducing topical drug waste in ophthalmic surgery.^36^ Third, it was a single-center study conducted in an academic hospital that handles more complex cases, requiring more resources and longer duration of surgeries, and may have already optimized certain practices. At the same time, academic settings may also involve additional procedures or inefficiencies not present in high-volume nonacademic centers. In addition, the academic hospital in which the study was performed serves a broad geographical area. Although variation in travel distances between different regions may lead to limited generalizability, by including patient travel movements within the study boundaries, we demonstrate that travel movements can play a significant role in the environmental impact of a care pathway. Our sensitivity analysis indicated that the carbon footprint could vary by −13% to +13% if patient and employee travel distances are 30% shorter or longer than those used in the main analysis.

In conclusion, the main environmental hotspots of cataract surgery are patient travel and disposable products. The most effective mitigation strategy to reduce the carbon footprint is implementing ISBCS. Additional mitigation strategies include reducing the use of disposable products, encouraging sustainable travel options, and switching to renewable energy. Together, these strategies offer a pathway to minimizing the environmental footprint of cataract surgery without compromising patient outcomes.

**WHAT WAS KNOWN**

- Cataract surgery is a high-volume procedure with a significant environmental impact, but studies on the environmental impact of cataract surgery focused mainly on carbon footprint.
- Previously published estimates of the carbon footprint of cataract surgery vary widely because of differences in methodology, making comparisons difficult.
- No study had systematically mapped the main environmental hotspots or tested targeted mitigation strategies.

**WHAT THIS PAPER ADDS**

- A life cycle assessment identified disposables and patient travel as the largest contributors to the environmental impact of cataract surgery.
- Immediate sequential bilateral cataract surgery and simplifying surgical kits are effective mitigation strategies.
- A comparison of process-based and monetary value-based methods showed a fourfold difference in impact estimates, highlighting the need for standardized assessment methods.

## AI Use

During the preparation of this work the authors used ChatGPT to improve readability and to correct language errors. After using this tool/service, the authors reviewed and edited the content as needed and take full responsibility for the content of the publication.
